# Functional and Morphological Adaptations in the Heart of Children Aged 12–14 Years following Two Different Endurance Training Protocols

**DOI:** 10.3390/sports11080157

**Published:** 2023-08-16

**Authors:** Lefteris Rafailakis, Chariklia K. Deli, Ioannis G. Fatouros, Athanasios Tsiokanos, Dimitrios Draganidis, Athanasios Poulios, Dimitrios Soulas, Athanasios Z. Jamurtas

**Affiliations:** Department of Physical Education and Sport Science, University of Thessaly, 42100 Trikala, Greece; lrafailakis@gmail.com (L.R.); delixar@pe.uth.gr (C.K.D.); ifatouros@uth.gr (I.G.F.); atsiokan@uth.gr (A.T.); ddraganidis@uth.gr (D.D.); dsoulas@pe.uth.gr (D.S.)

**Keywords:** exercise, cardiac, track and field, athlete, anaerobic threshold, VO_2_max

## Abstract

This study investigated the cardiac functional and the morphological adaptations because of two endurance training protocols. Untrained children (N = 30, age: 12–14 years) were divided into three groups (N = 10/group). The first group did not perform any session (CONTROL), the second performed ventilatory threshold endurance training (VTT) for 12 weeks (2 sessions/week) at an intensity corresponding to the ventilatory threshold (VT) and the third (IT) performed two sessions per week at 120% of maximal oxygen uptake (VO_2_max). Two other sessions (30 min running at 55–65% of VO_2_max) per week were performed in VVT and IT. Echocardiograms (Left Ventricular end Diastolic Diameter, LVEDd; Left Ventricular end Diastolic Volume, LVEDV; Stroke Volume, SV; Ejection Fraction, EF; Posterior Wall Thickness of the Left Ventricle, PWTLV) and cardiopulmonary ergospirometry (VO_2_max, VT, velocity at VO**_2_**max (vVO_2_max), time in vVO_2_max until exhaustion (Tlim) was conducted before and after protocols. Significant increases were observed in both training groups in LVEDd (VTT = 5%; IT = 3.64%), in LVEDV (VTT = 23.7%; ITT = 13.6%), in SV (VTT = 25%; IT = 16.9%) but not in PWTLV and EF, after protocols. No differences were noted in the CONTROL group. VO**_2_**max and VT increased significantly in both training groups by approximately 9% after training. Our results indicate that intensity endurance training does not induce meaningful functional and morphological perturbations in the hearts of children.

## 1. Introduction

It is well-known that physical training is associated with hemodynamic changes that bring about morphological and functional adaptations in the heart of athletes [1,2,3,4,5]. The increase in the heart mass of athletes was first observed by Henschen and Skidlauf in 1899 [6] through a percussion-based examination of the thorax, and was later confirmed through radiography and proven through autopsy. The advent of echocardiography allowed researchers to obtain a more detailed insight into the adaptations that a heart undergoes through exercise, while the use of magnetic resonance imaging (MRI) in recent years has further helped elucidate these adaptations. Although the existence of an “athletic heart” is nowadays commonly accepted among researchers, the Morganroth theory [6], which prevailed for many years, is verified only in part, since the concentric cardiac adaptation of dynamic-sport athletes and the non-increase in the wall thickness of the left ventricle (LV) in endurance athletes of strenuous sports are not verified by more recent research [7,8,9]. Concentric hypertrophy is associated with increased left ventricular wall thickness without overall enlargement and increased Left Ventricular end Diastolic Diameter (LVEDd), whereas eccentric hypertrophy is characterized by dilatation of the ventricular chamber, accompanied by symmetrically increased wall thickness. All athlete categories show an increase in LVEDd and the Posterior Wall Thickness of the Left Ventricle (PWTLV), which is analogous. However, in endurance sports, cardiac hypertrophy is most evident with long-distance athletes exhibiting extremely high values in LVEDd [3,10,11,12].

The literature contains several studies examining the effect of endurance training on the morphology and function of the adult heart [9,13,14,15,16,17], but few have addressed the effects of endurance training on the hearts of children and adolescents [18,19,20,21,22,23]. Previous studies on preadolescent (11–13 years of age) distance runners indicated that endurance training does not result in significant changes in cardiac remodeling [24], whereas five months of swimming training resulted in significant remodeling of the right ventricle of competitive athletes [25]. A comparison between preadolescent cross country skiers and non-competing individuals revealed that adaptive remodeling can occur in this age since athletes had greater left ventricular mass and greater left and right ventricular chamber dimensions [26]. Furthermore, a shift in the development of the young athlete’s heart was reported until the age of 15. Active endurance athletes underwent eccentric remodeling, whereas a dynamic switch to concentric remodeling took place between the ages of 15 to 18 years of age [22]. However, it is not clear as to what the effect on the morphology and function of the child’s heart would be due to different training approaches, including ones that incorporate intense training.

The training load on young endurance athletes is an important unanswered question. The increasing competitiveness in sports has led to a trend of athletes training for endurance events at younger and younger ages. This shift has led to more intense training regimes, including long-distance running, swimming, and bike rides being incorporated into the regular routines of younger athletes looking to compete in these disciplines. Additionally, athletes are also being pushed to compete in multiple endurance events, when previously only one or two were the norm. The quantity and intensity that should be included in the training of young athletes, so that the development of their heart is optimized creating conditions for a healthy, strong heart in the future and hence better performance, is a point that needs addressing. The notion that intense training at an early age could cause cardiac limitations so that the future of the child in sports would be jeopardized is also of great interest and with no firm answers so far. It is important to assess young athletes regularly for their cardiac adaptations due to training in order to identify whether parameters of training, like volume and intensity, result in detrimental physiological and/or structural changes. We are not aware of any study that examined the effects of different training approaches on the morphological and functional aspects of a child’s heart. Therefore, the first aim of this study was to investigate the cardiac functional and morphological adaptations, as well as the performance changes of children aged 12 to 14 brought about by two endurance training protocols. Furthermore, in order to assess whether morphological and functional development of the heart precedes, and performance improvement follows with some delay, a correlation analysis was performed between performance, functional and morphological characteristics following endurance training.

## 2. Material and Methods

### 2.1. Participants

**Figure 1:**
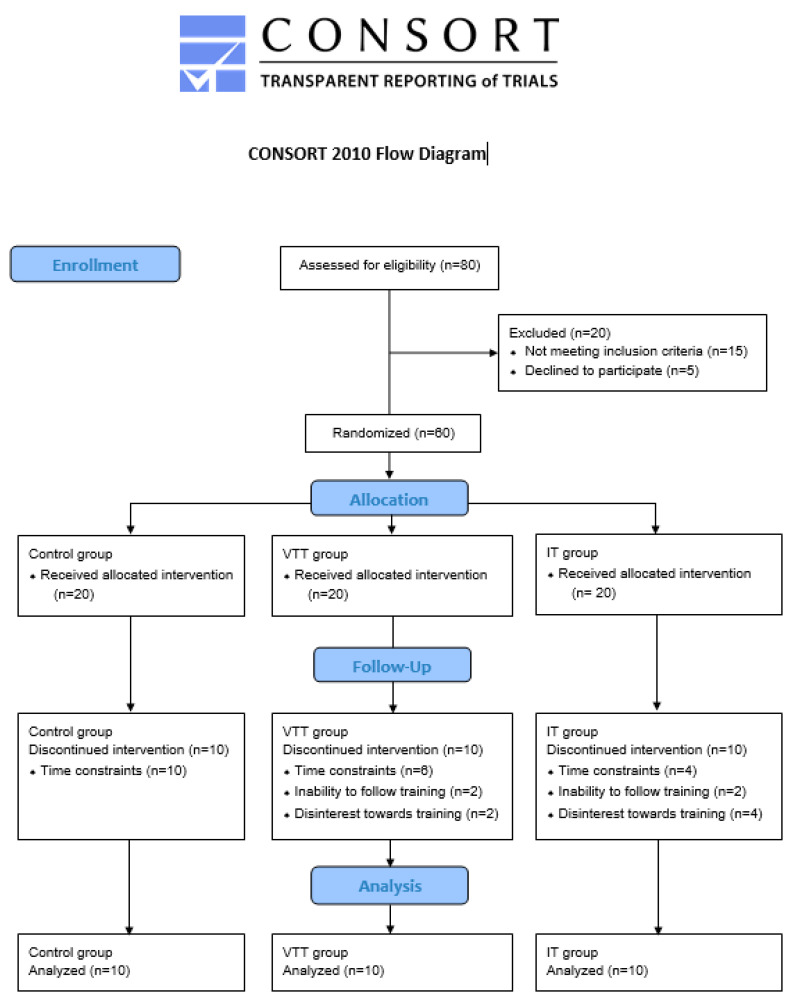
CONSORT flow chart.

Thirty healthy untrained children (16 boys and 14 girls) participated and were randomly divided into 3 groups. We used the sealed envelope method of randomization to eliminate any potential bias and to ensure that the results of the trial are free from bias and accurately reflect the effectiveness of the intervention studied herein. The first group consisted of 5 girls and 5 boys (Age = 12.4 ± 0.7 years), served as the control group and did not follow a structured training protocol. The second group consisted of 6 girls and 4 boys (Age = 12.8 ± 0.6 years) and these participants trained at an intensity corresponding to the ventilatory threshold (ventilatory threshold training group, VTT): an intensity that is considered moderate. The third group consisted of 3 girls and 7 boys (Age = 13.1 ± 0.3 years) and trained at 120% of VO_2_max (intensity training group, IT): an intensity that is considered highly intense. The rationale behind choosing these two intensities was to assess whether the intense training would lead to compromised cardiac remodeling. Figure 1 shows the CONSORT diagram of the study.

Inclusion criteria for the participants were: (1) Healthy, 12–14 years of age, and (2) not participating in an organized sporting activity. Furthermore, none of the participants were on any prescribed medication and were able to cope with the strain of the experimental procedure. All participants and their parents declared their consent both orally and in writing after they were informed about the investigation process. Four participants dropped out because they could not follow the training (2 in VTT and 2 in IT), 6 participants developed lack of interest towards training (2 in VTT and 4 in IT) and 20 participants stopped training due to time constraints (6 in VTT and 4 in IT). No injuries were reported during the training period. The procedures followed were in full agreement with the Declaration of Helsinki of 1975, as it was revised in 2000, while an approval by the Ethics Committee of the University was provided (protocol number 505).

### 2.2. Experimental Design

**Figure 2:**
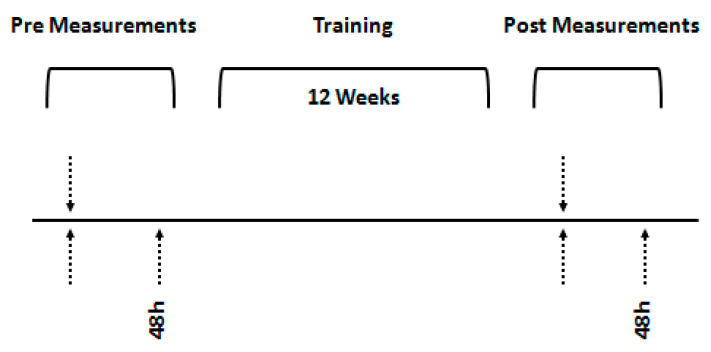
Schematic representation of the experimental design. Upward arrows indicate cardiopulmonary exercise measurements and downwards arrows indicate echocardiographic measurements.

Participants visited the laboratory two times prior to the training intervention. During the first visit, the anthropometric characteristics and their biological age was recorded using the Tanner method, an echocardiographic study was conducted, their maximal oxygen uptake (VO**_2_**max) was measured and their ventilatory threshold (VT) was determined. During the second visit, the time in velocity at VO_2_max (vVO_2_max) until exhaustion (Tlim) was determined. Measurements were obtained with a difference of at least 48 h. The same procedures were followed after the training intervention. Participants were instructed to refrain from participation in physical activity or exercise 48 h before the measurements. The research design is shown in Figure 2.

### 2.3. Anthropometric Measures

Body mass, height and fat percentage of seven skinfolds were evaluated. All the anthropometric assessments were performed according to the instructions of the American College of Sports Medicine (ACSM, 9th Edition) in the morning, following an overnight fast. Body mass was measured to the nearest 0.05 kg (Seca alpha 770 scales, Vogel & Halke Hamburg, Germany) while the participants were lightly dressed and barefoot. Standing height was measured to the nearest 0.5 cm (Seca Stadiometer 208; Vogel & Halke Hamburg, Germany). Percentage body fat was estimated from 7 skinfold-thickness measures (average of two measurements of each site) by using a Harpenden caliper (John Bull, St. Albans, United Kingdom). The Siri skinfold-thickness equation was used to calculate body fat (ACSM, 9th Edition). The biological age of the participants was assessed in the lab by a trained medical examiner of the participants’ Tanner’s sexual maturation stages.

### 2.4. Maximal Oxygen Uptake

Maximal oxygen uptake (VO_2_max) was assessed through the use of a protocol of gradual speed increase on a treadmill (TechnoGym Run Race, Rome, Italy). The procedure was carried out after a 12 min warm-up (8 min of relaxed jogging at 60% of maximal heart rate, and 4 min of stretching). The initial speed was set to 8 km·h^−1^. Then, increased by 1 km·h^−1^ for every one minute. The O_2_ and CO_2_ rates in the exhaled air were measured through a gas analyzer (VO2000, Sensormedics, Yorba Linda, CA, USA), which was calibrated before each test using standard gases of known concentration, while the heart rate was recorded with a heart-rate monitor (Polar S410, Kempele, Finland). The volumes of exhaled gases were recorded (breath by breath) every 20 s. Criteria used to determine VO_2_max were: (i) participants’ exhaustion, (ii) a <2 mL·kg^−1^·min^−1^ increase in VO_2_ with an increase in work rate, (iii) a respiratory exchange ratio greater than or equal to 1.10, (iv) a heart rate within 10 bpm of the theoretical maximum heart rate (220-age). The velocity at maximal oxygen consumption (vVO_2_max) and the velocity at ventilatory threshold (VT) were recorded. The VT was determined by the V-slope method [27], while vVO_2_max was defined according to Billat et al. [28].

### 2.5. Time to Exhaustion

In order to determine time to exhaustion (retention time in vVO**_2_**max until exhaustion, Tlim) participants, had to reach their individual vVO**_2_**max, in 30 s after starting their examination from a state of inertia. Once a subject reached the vVO**_2_**max, the measurement of Tlim began. Timing lasted until the subject was volitionally exhausted.

### 2.6. Echocardiogram

The echocardiography examinations were conducted by a registered diagnostic medical sonographer with more than five years experience, using the clinical imaging system (Hitachi Aloka ProSound Alpha 6, Hitachi Aloka Medical Ltd., Tokyo, Japan) and 5-MHz transducer. Standard echocardiographic views (parasternal, apical, and substernal) were obtained. All images were reviewed to confirm that parasternal long axis (PSLA) parasternal short axis (PSSA), four-chamber (4-CH), and two-chamber (2-CH) views were acquired with at least three complete cardiac cycles, and images were obtained at a frame rate of 50 to 80 frame/s. 2-CH and 4-CH images were collected and yielded comparable results. However, due to time limitations imposed by the children, it was not possible to acquire these images from all participants. All the recorded views from the parasternal long axis (PSLA) were reviewed to select the most suitable with minimal heart rate variability. Guidelines by the European association of cardiovascular imaging were adhered to when acquiring and analyzing the recorded images. The sonographer was blinded to the characteristics of the participants, the group allocation and time point of measurement.

### 2.7. Training Protocol

The training period for both training groups was 12 weeks. All training sessions were supervised. The participants engaged in four workouts each week, with two of them being the same between the two training groups, and consisted of running for 30 min at 55–65% of vVO**_2_**max. The other two workouts started with a warm-up which consisted of a 10 min jog and 10 min of stretching exercises and then the participants in the VT group (Table 1) ran for 20 min at the speed corresponding to their VT, while those in the IT group (Table 2) ran three sets of 200–150–100 m at an intensity corresponding to 120% of vVO**_2_**max. The rest interval between sets was two minutes. There was no difference in the total training time for the two groups. After the fourth week, the intensity was increased for both groups by about 5%. The same increase was applied after the eighth week. The control group participated for 45 min twice per week in the physical education classes at school. Participants were advised not to engage in any additional structured exercise program except the physical education classes.

### 2.8. Statistical Analysis

The normality of all dependent variables was assessed by the Kolmogorov–Smirnov test and was found not to differ significantly from the norm. Initially, a one-way analysis of variance was performed to test whether there were differences between the three groups on Tanner variable. The results showed that there was a statistically significant difference, and therefore, a two-way (group × time) analysis of covariance (ANCOVA) for repeated measurements was carried out, using the Tanner variable as covariant. LSD post hoc test was used to check for possible differences between and within groups. Pearson’s correlation coefficient (*r*) was calculated using the measurements following the exercise training so as to examine the relationship of SV, VO**_2_**max, LVEDd, LVEDV and PWTLV. Significance was set at *p* < 0.05. Analysis was performed using the SPSS 26 software (IBM SPSS Statistics 26).

## 3. Results

There were no adverse events during the study. Compliance to the training was 95% for the VTT group and 96% for the IT group. A significant interaction between groups and time was found for body weight (F2,26 = 3.33, *p* = 0.05). A significant increase between pre and post measurements for body weight (F1,26, *p* < 0.01) was found in the control group (Table 3). No significant interaction between groups and time was found for height (F2,26 = 0.25, *p* = 0.78). Furthermore, there were no significant differences between pre and post measurements in any of the three groups (Table 3). A significant interaction between groups and time was found for % body fat (F2,26 = 6.71, *p* = 0.004). A significant decrease between pre and post measurements for % body fat was found in the VTT group (F1,26, *p* < 0.001) and in the IT group (F1,26, *p* < 0.05, Table 3).

A significant interaction between groups and time was found for LVEDd (F2,26 = 6.75, *p* = 0.004). A significant increase between pre and post measurements was found for LVEDd in the VTT group (F1,26, *p* < 0.001) and in the IT group (F1,26, *p* < 0.001, Table 4). A significant interaction between groups and time was found for LVEDV (F2,26 = 11.65, *p* = 0.000). A significant increase between pre and post measurements was found for LVEDV in the VTT group (F1,26, *p* < 0.001) and in the IT group (F1,26, *p* < 0.001, Table 4). No significant interaction between groups and time was found for PWTL (F2,26 = 1.05, *p* = 0.37). A significant interaction between groups and time was found for SV (F2,26 = 8.06, *p* = 0.002). A significant increase between pre and post measurements was found for SV in the VTT group (F1,26, *p* < 0.001) and in the IT group (F1,26, *p* < 0.001, Table 4). No significant interaction between groups and time was found for height (F2,26 = 2.45, *p* = 0.11). Furthermore, there were no significant differences between pre and post measurements in any of the 3 groups (Table 4).

No significant interaction between groups and time was found for MHR (F2,26 = 1.14, *p* = 0.34). Furthermore, there were no significant differences between pre and post measurements in any of the three groups (Table 5). No significant interaction between groups and time was found for VO_2_max (F2,26 = 1.26, *p* = 0.30 In contrast, there was a significant decrease between pre and post measurements for VO_2_max in the VTT group (F1,26, *p* < 0.001) and in the in the IT group (F1,26, *p* < 0.001, Table 5). No significant interaction between groups and time was found for VT (F2,26 = 0.89, *p* = 0.42). There was a significant decrease between pre and post measurements for VT in the VTT group (F1,26, *p* < 0.001) and in the in the IT group (F1,26, *p* < 0.001, Table 5). No significant interaction between groups and time was found for Tlim (F2,26 = 1.34, *p* = 0.28). Furthermore, there were no significant differences between pre and post measurements in any of the 3 groups (Table 5). No significant interaction between groups and time was found for vVO_2_max (F2,26 = 1.72, *p* = 0.20). Furthermore, there were no significant differences between pre and post measurements in any of the three groups (Table 5).

### Correlation Analysis

A correlation analysis was applied utilizing the combined data from the three groups in order to examine the relationship between the SV, VO_2_max, LVEDd, LVEDV, and PWTLV variables in the second measurement. A strong correlation was based on a correlation coefficient, ranging from −0.7/+0.7 to −0.9/+0.9; a moderate correlation was based on a correlation coefficient, ranging from −0.4/+0.4 to −0.6/+0.6; and a weak correlation was based on a correlation coefficient, ranging from −0.1/+0.1 to −0.3/+0.3 [29]. The results (Table 6) showed that there was a moderately positive correlation between SV and VO_2_max. There was also a strong positive correlation between the variables of SV and LVEDd and LVEDV, while there was no statistically significant correlation between the SV and PWTLV. The VO_2_max positively correlated with LVEDd, LVEDV and PWTLV. Furthermore, a strong positive correlation was found between LVEDd and LVEDV while there was no statistically significant correlation between LVEDd and PWTLV. Finally, no statistically significant correlation was found between LVEDV and PWTLV.

## 4. Discussion

### 4.1. Summary

This study was undertaken in order to investigate the cardiac functional and morphological adaptations, as well as the performance changes that would be brought about as a result of endurance training in untrained children who are at a developing age (12–14 years old). As children mature and go through puberty, physical development might be different and early maturation might have an effect on function and morphology of the heart as well as athletic performance. In our study, even though a randomization process took place, the assessment of biological age through the Tanner scale revealed significant differences between the groups. To eliminate this effect, an analysis of covariance was performed with Tanner as a covariate. The two different endurance training protocols led to similar changes after 12 weeks of training in both the morphology and function of the heart and the data defining the performance of the participants.

As expected in accordance with the Frank-Starling law, the LVEDd, LVEDV and SV increased significantly. Furthermore, there was a significant increase in VO_2_max, and VT. The increase in LVEDd was not accompanied by an increase in PWTLV, while the hearts of the participants appeared to develop eccentrically following both training protocols. It seems that morphological changes of the heart were followed by positive changes in the parameters that determine the performance of young athletes.

### 4.2. Performance

VO_2_max, vVO_2_max, VT and Tlim increased as a result of training. VO**_2_**max and VT was increased by approximately 9% in both training groups, whereas the increase in both parameters was by only 3% in the control group, probably due to participation in the School’s physical education classes. Previous work has shown that aerobic training in young girls did not elicit significant changes in aerobic performance [30]. However, more recent studies indicate that aerobic training at this age does result in significant changes in peak VO_2_ [31] with a meta-analysis suggesting an increase between 5–6% in children [32]. Our results for VO**_2_**max indicate a higher increase from the aforementioned typical changes suggested in the previous meta-analysis [32]. It seems that the duration and intensity of training played an important role in producing these results.

### 4.3. Morphological Adaptations

LVEDd increased significantly in both experimental groups as a result of training, while PWTLV showed no differences in either group. These results are in line with previous studies, in which it was observed that at the beginning of a sporting career, LVEDd appears to increase without an increase in PWTLV, which in turn may happen at the age of 9–12 years [13,20,21,24,33,34] or a little later at the age of 12 to 14 [22,31]. The most pronounced increase in LVEDd, especially in endurance athletes, is justified by the mere finding that there is an increased blood requirement in aerobic forms of exercise. That increased demand for blood leads to increased venous return, and therefore, a greater preload, which in turn will result in an increased stroke volume by the left ventricle, imposed by the Frank-Starling mechanism [35,36]. This is potentially the main reason that LVEDd increases earlier than the thickness of the LV wall. The delayed increase in the PWTLV is also explained by inadequate hormonal support, especially by the lack of testosterone in younger age groups, which is necessary for the growth of the heart wall. It is reported that hormonal changes lead to heart muscle increases after the age of 13–14 years [37,38].

Between the two experimental groups, there were no significant differences in LVEDd and PWTLV. This finding suggests that the two different training protocols resulted in similar morphological adaptations in the left ventricle of the participants. In a similarly contacted study by Obert et al. [34], 29 boys and girls of 10 to 11 years of age who participated in three training workouts a week for a period of 13 weeks, in which they ran at an intensity of >80% of the maximum heart rate. The results of this study are similar to ours, since LVEDd increased significantly (+4.6%), while a reduction in PWTLV (−10.7%) was observed as a result of endurance training [34]. Associations between remodeling of the right ventricle due to exercise training was reported also [39].

### 4.4. Functional Adaptations

LVEDV and SV increased significantly after the training period in both experimental groups. The results are in accordance with the Frank-Starling law since the increase in LVEDd results in increased LVEDV and, consequently, of SV. Also, as it was expected, there was no increase in the EF, since there is no data to indicate that EF changes as a result of endurance training. Between the two experimental groups, there were no significant differences in LVEDd, LVEDV, SV and EF, so it would appear that, on a functional level, there were similar adaptations as a result of the two different types of training.

### 4.5. Correlation of Cardiovascular and Ergospirometric Data

The increases in LVEDd, LVEDV and SV would seem to result in an increase in VO_2_max, which was verified (VO_2_max increased in both groups by 9%). Our hypothesis was that there would be a linear correlation of LVEDd, LVEDV and SV with VO_2_max. Overall, these data suggest that the heart undergoes a morphological and functional development as age increases, which leads to significant performance improvements.

VO_2_max was positively correlated with LVEDd, LVEDV and SV, but not with PWTLV at the completion of the training courses of both groups. It seems that the heart of the children developed eccentrically, and that improved performance came mainly from the increase in LVEDd and LVEDV, which in turn leads to a higher SV and VO_2_max without affecting the PWTLV. Peripheral factors such as mitochondria number and density, enzyme concentration and activity and capillary density play a significant role on aerobic performance [40]. Changes in these factors could play a significant role on the timing of performance increases due to training at this age, and even though the Tanner method was used to adjust for the effects of age, the lack of assessment of these factors constituted toward a limitation of the study, and it is something that should be determined in future research.

Our initial assumption, based on the Morganroth theory, was that intensive training would lead to concentric heart development, as opposed to a less intense workout, which would cause eccentric development [41]. If our assumption was verified, then that would create a restrictive environment within the heart through a concentric heart development that would reduce the chances for improvement in performance in later life. It was reported that endurance training leads to a more eccentric development of the heart, whereas intense exercise, like weight training, induces a more concentric development of the heart [2,42], mainly due to increased wall thickness induced by pressure overload [43]. The results from this study do not seem to accept this assumption, despite the opposite views presented previously [41]. Undoubtedly, it should be noted that it cannot be ruled out that a more intensive training and/or greater time span could have different effects. Moreover, Morganroth et al. [41], in their paper, examined adults, and their findings are not fully compatible and comparable with the ones from the present study. Data comparison with other similar studies cannot be conducted since there are no comparable research projects, so we came up with some theories based on plausible assumptions. The two types of training appear to affect the heart morphologically and functionally in a similar way.

One of the biggest enigmas in endurance training is the reason young athletes do not have the development that their performance at an early age predisposes. It is very common that young endurance athletes achieve outstanding performances and later reach a performance plateau, which results in their discontinuation of participation in the sport (burn out-drop out) [44,45]. A reasonable assumption is that in cases of high-training loads at an early age, the heart grows concentrically with an excessive increase in PWTLV, which is not accompanied by a corresponding increase in LVEDd and LVEDV, thus resulting in structural limitations at an older age. If this was indeed the case, it would most likely be the main reason why young endurance athletes exhibit a plateau or decline in performance at older ages, since the increase in PWTLV would hinder growth in LVEDd and LVEDV. Therefore, in accordance to the Frank-Starling law, SV would be impossible to increase, and, as a consequence, could prevent an increase in VO_2_max. This does not seem to be confirmed by the present study, although it requires more investigation at greater intensities and for periods longer than 12 weeks.

Our initial hypothesis was that the IT group would show even a small concentric tendency in comparison to the VTT group, and greater increases in performance parameters, but this did not happen. Participants took part in two different training programs with the main difference in these being only the intensity of exercise. The frequency (4 times a week), duration (180 min a week) and the volume (started with 21 km a week and ended with 27 km a week) of training was the same between groups whereas the intensity in two training sessions was almost twice in the IT group compared to VTT group. Even with this distinct difference in the intensity, the two groups did not differentiate in exercise performance, functional and morphological indices of the left ventricle of the heart. It seems that the pressure overload induced by different type of intense activities, like weight training [42], has a different effect on the left ventricle compared to running, which is a more rhythmic type of exercise. Therefore, it seems that the intensity of exercise does not appear to result in significant perturbations in the functional and morphological characteristics of the heart of a young child and is not a main constraint in the development of a young endurance athlete.

### 4.6. Limitations

As it was mentioned previously, the lack of assessment of peripheral factors contributing to enhancement of exercise performance constitutes a limitation of the study. Furthermore, absence of assessment of important hormones contributing to the development of a child and affecting performance and duration of the training program constitute additional limitations. Longer duration and different intensities in the training programs could potentially lead to enhancement in performance along with the adaptations found in the heart of the young children, and this is something for future research to determine.

## 5. Conclusions

In conclusion, the two different endurance training protocols led to similar changes in morphological and functional characteristics of the heart of untrained children and to similar enhancements in exercise performance following a period of 12 weeks. Thus, from these results, it could be assumed that the Frank-Starling law is verified, since exercise increased LVEDd and LVEDV, and, as a result, SV increased. Future studies should examine longer training duration programs and assess whether hormonal and peripheral factors could potentially affect exercise performance in this age group.

## Figures and Tables

**Table 1:** Exercise training protocol—Ventilatory Threshold group.

Week	Intensity		Duration	Volume	Frequency	
	Same for the Training Groups	Training	(min)	(km)	Same for the Training Groups	Training
1	55–60% vVo_2_max	@Ventilatory threshold	180	21	2	2
2			180	22	2	2
3			180	22	2	2
4			180	23.5	2	2
5	Increase 5% vVo_2_max	Increase 5% vVo_2_max	180	24	2	2
6			180	24.5	2	2
7			180	24.5	2	2
8			180	25.5	2	2
9	Increase 5% vVo_2_max	Increase 5% vVo_2_max	180	26	2	2
10			180	26	2	2
11			180	26.5	2	2
12			180	27	2	2

**Table 2:** Exercise training protocol—Intensity group.

Week	Intensity		Duration	Volume	Frequency	
	Same for the Training Groups	Training	(min)	(km)	Same for the Training Groups	Training
1	55–60% vVo_2_max	200–150–100 mRest 2 min120 vVO_2_max	180	21	2	2
2			180	22	2	2
3			180	22	2	2
4			180	23.5	2	2
5	Increase 5% vVo_2_max	Increase 5% vVO_2_max	180	24	2	2
6			180	24.5	2	2
7			180	24.5	2	2
8			180	25.5	2	2
9	Increase 5% vVo_2_max	Increase 5% vVO_2_max	180	26	2	2
10			180	26	2	2
11			180	26.5	2	2
12			180	27	2	2

**Table 3:** Participants’ Personal characteristics before and after the training period.

Variable	CONTROL		VTΤ		IT	
	Before	After	Before	After	Before	After
Age (years)	12.4 ± 0.7		12.8 ± 0.6		13.1 ± 0.3	
Body Mass (kg)	39.9 ± 5.8	40.8 ± 6.3 **	40.5 ± 4.6	40.4 ± 4.5	47.7 ± 5.5	47.6 ± 5.5
Height (cm)	157.1 ± 4.3	157.4 ± 4.4	156.2 ± 4.6	156.8 ± 4.5	159.2 ± 4.9	159.4 ± 4.5
Fat (%)	13.9 ± 3.9	14.3 ± 4.1	16.2 ± 3.0	15.0 ± 3.9 ***	15.0 ± 4.6	14.2 ± 3.9 *

* Sig diff vs. before within the same group (*p* < 0.05); ** Sig diff vs. before within the same group (*p* < 0.01); *** Sig diff vs. before within the same group (*p* < 0.001); VTΤ: ventilatory threshold group; IT: intensity group.

**Table 4:** Functional and morphological characteristics of the heart of the participants before and after the training period.

Variable	CONTROL		VTT		IT	
	Before	After	Before	After	Before	After
LVEDd (mm)	43.7 ± 4.6	43.5 ± 4.6	44.1 ± 2.5	46.5 ± 2.0 ***	43.9 ± 2.6	45.5 ± 2.7 ***
LVEDV (mL)	88.1 ± 21.4	89.6 ± 22.4	81.3 ± 10.2	100.3 ± 12.5 ***	83.8 ± 13.2	95.0 ± 12.3 ***
PWTLV (mm)	8.15 ± 1.05	8.25 ± 1.03	8.00 ± 1.02	8.40 ± 1.02	8.15 ± 0.97	8.70 ± 1.25
SV (mL)	59.6 ± 16.0	60.4 ± 15.6	60.7 ± 9.2	75.9 ± 9.6 ***	57.5 ± 9.4	67.3 ± 10.3 ***
ΕF (%)	69.10 ± 5.66	70.10 ± 5.97	72.40 ± 6.1	75.30 ± 0.56	72.20 ± 6.81	71.50 ± 5.38

*** Sign. Diff. vs. Before in the same group (*p* < 0.001); LVEDd: Left Ventricular end Diastolic Diameter; LVEDV: Left Ventricular end Diastolic Volume; PWTLV: Posterior Wall Thickness of the Left Ventricle; SV: Stroke Volume; EF: Ejection Fraction; VTT: ventilatory threshold training group, IT: intensity training group,

**Table 5:** Performance characteristics of the participants’ before and after the training period.

Variable	CONTROL		VTT		IT	
	Before	After	Before	After	Before	After
MHR (bpm)	206.2 ± 3.8	205.6 ± 3.6	205.0 ± 4.6	205.2 ± 4.9	206.2 ± 3.9	203.9 ± 4.9
VO_2_max (mL/kg/min)	46.8 ± 6.54	48.6 ± 7.37	46.0 ± 6.85	50.2 ± 8.46 ***	48.7 ± 5.98	53.2 ± 6.88 ***
VT (mL/kg/min)	36.13 ± 7.14	37.53 ± 7.96	35.05 ± 6.96	37.99 ± 8.48 ***	37.57 ± 6.26	41.15 ± 6.73 ***
Tlim (min)	3.35 ± 0.71	3.79 ± 0.77	3.59 ± 0.81	3.63 ± 1.15	4.63 ± 1.44	4.04 ± 1.29
vVO_2_max (km/h)	14.10 ± 0.93	14.70 ± 0.94	13.95 ± 0.98	14.75 ± 1.13	14.15 ± 1.52	14.31 ± 1.88

*** Sign. Diff. vs. Before in the same group (*p* < 0.001); MHR: maximum heart rate; VO_2_max: maximal oxygen uptake; VT: ventilatory threshold; RE: running economy; Tlim: T limit; vVO_2_max: velocity at VO_2_max; VTΤ: ventilatory threshold group; IT: intensity group

**Table 6:** Correlation analysis between variables SV, VO**_2_**max, LVEDd, LVEDV and PWTLV in the second measurement (combined data).

	1	2	3	4
1. SV	-			
2. VO_2_max	0.544 **	-		
3. LVEDd	0.830 **	0.505 **	-	
4. LVEDV	0.897 **	0.656 **	0.868 **	-
5. PWTLV	0.204	0.436 *	0.213	0.167

SV: Stroke Volume; LVEDd: Left Ventricular end Diastolic Diameter; LVEDV: Left Ventricular end Diastolic Volume; PWTLV: Posterior Wall Thickness of the Left Ventricle * *p* < 0.05, ** *p* < 0.001.

## Data Availability

The data present in this study are available upon request from the corresponding author. The data are not publicly available due to privacy restrictions.
